# Unveiling urethral cellular heterogeneity in menopause through single-nucleus RNA sequencing

**DOI:** 10.3389/fphys.2026.1753680

**Published:** 2026-03-02

**Authors:** Jinghao Mu, Jian Xiong, Shunchang Zhou, Zhenliang Qin, Jianlin Chen, Hui Guo, Guanghui Du

**Affiliations:** 1 Department of Urology, Tongji Hospital, Tongji Medical College, Huazhong University of Science and Technology, Wuhan, Hubei, China; 2 Center of Experimental Animals, Huazhong University of Science and Technology, Wuhan, Hubei, China; 3 Institute of Organ Transplantation, Tongji Hospital, Tongji Medical College, Huazhong University of Science and Technology, Wuhan, Hubei, China; 4 Key Laboratory on Transplantation of Ministry of Education and Ministry of Health, Wuhan, Hubei, China

**Keywords:** epithelial cells, fibroblasts, menopause, rats, snRNA-seq, urethra

## Abstract

**Background:**

Estrogen homeostasis is crucial for the structure and function of the urethra, and estrogen deprivation resulting from menopause, ovariectomy, or ovarian dysfunction may lead to various urethral dysfunctions. However, the specific molecular mechanisms involved are still not fully understood.

**Methods:**

Urethras from three ovariectomized (OVX) rats and three Sham rats were collected for snRNA-seq analysis. Data analysis included unsupervised clustering using the UMAP algorithm to identify distinct cell types based on marker gene expression. Differential gene expression analysis was performed to identify changes in estrogen-related gene expression across different cell types. Functional enrichment analysis was conducted to elucidate biological pathways associated with differentially expressed genes. Additionally, cellular interactions and developmental trajectories were analyzed to characterize cellular dynamics during menopause.

**Results:**

Here, we profiled 69,529 single-nucleus transcriptomes from rat urethra (three OVX rats and three Sham rats). The snRNA-seq analysis revealed pronounced cellular heterogeneity and menopause-associated transcriptional reprogramming. We identified Fos as a key transcription factor associated with epithelial cell communication and differentiation under estrogen-deprived conditions. In addition, basal epithelial cells displayed EMT-associated transcriptional programs and a potential epithelial-to-mesenchymal continuum toward a mesenchymal-like state in OVX rats. We also identified Tmem233 as a hub gene in a striated muscle contraction-related module enriched in type IIa myofibers, and observed heightened inflammatory activation in immune cells, particularly T cells, in OVX rats.

**Conclusion:**

In summary, our study provides a comparative analysis of the snRNA-seq data from the urethra of female rats, elucidating cellular and molecular changes during menopause.

## 1 Introduction

Menopause is a major physiological transition in women, marked by the cessation of ovarian function and a sustained decline in estrogen levels (Alperin et al., 2019). Estrogen deficiency induces atrophic and degenerative changes in the lower urinary tract and is linked to a spectrum of urinary symptoms, including urgency, frequency, dysuria, recurrent urinary tract infections, and stress urinary incontinence (Robinson et al., 2013). Urethral mucosal thinning, extracellular matrix remodeling, reduced vascularity, and weakening of smooth/striated muscle together impair urethral closure and barrier functions (Robinson et al., 2013; Andersson and Uvelius, 2024; Zhang et al., 2024). Despite the clinical burden, the cell-type-specific molecular programs and intercellular signaling networks that mediate urethral remodeling in menopause remain incompletely defined.

Current management of postmenopausal lower urinary tract symptoms relies on behavioral therapy/pelvic floor muscle training, local estrogen supplementation, and surgical or bulking procedures; however, therapeutic responses are variable, mechanisms are not fully understood, and there is no disease-modifying therapy that restores urethral structure and function (Alperin et al., 2019; Robinson et al., 2013; Wang et al., 2023). A comprehensive cellular atlas of the menopausal urethra could (i) pinpoint estrogen-responsive cell populations, (ii) reveal signaling pathways driving epithelial atrophy, fibrosis, and immune activation, and (iii) nominate cell- and pathway-level targets for regenerative or cell-based interventions (Wang et al., 2023).

Epithelial–mesenchymal transition (EMT) is a conserved cellular program through which epithelial cells acquire mesenchymal features, enabling migration and tissue remodeling (Thiery and Sleeman, 2006). EMT-like programs contribute to embryonic development and wound repair, whereas persistent activation is implicated in fibrosis and pathological remodeling (Kalluri and Weinberg, 2009; Zeisberg and Neilson, 2010). In the lower urinary tract, chronic estrogen deprivation may alter epithelial–stromal homeostasis and engage EMT-associated pathways. Estrogen (particularly 17β-estradiol) can modulate EMT through multiple signaling axes, including TGF-β and ERK1/2 (Mendoza et al., 2011; Moustakas and Heldin, 2009).

Single-nucleus RNA sequencing (snRNA-seq) has significantly advanced our understanding of cellular heterogeneity in complex tissues (Eraslan et al., 2022; Morabito et al., 2021; Zhuang et al., 2025). Unlike scRNA-seq, which requires intact viable single cells, snRNA-seq profiles isolated nuclei and is therefore well-suited for tissues that are difficult to dissociate without inducing cell loss or stress-related transcriptional artifacts (Kim et al., 2023). We prioritized snRNA-seq in this study because the urethra is a fibromuscular organ enriched in extracellular matrix and striated muscle, making enzymatic dissociation for scRNA-seq challenging and prone to biased recovery of certain cell types. Nuclei isolation enables robust capture of large/fragile cell types (e.g., striated muscle) and preserves transcriptional states in this context.

In this study, we generated a single-nucleus transcriptomic atlas of the female rat urethra under sham and ovariectomy-induced hypoestrogenic conditions. By integrating cell-type-resolved differential expression, cell–cell communication, and trajectory analyses, we delineate menopause-associated remodeling programs in epithelial, stromal, immune, and muscle compartments, providing a resource for mechanistic studies and potential therapeutic target discovery.

## 2 Materials and methods

### 2.1 Animal models

Female Sprague–Dawley rats (10–12 weeks, 220–260 g) were purchased from the Hubei BIONT Biological Technology Co., Ltd. Individually housed rats were kept in a specific-pathogen-free (SPF) facility with free access to food and water in a 12:12 light:dark cycle at 22 °C–26 °C and 45%–55% humidity. The ethics committee of Huazhong University of Science and Technology approved all animal procedures (Approval No. IACUC 4606). After 1 week of acclimation, rats were randomly assigned to the sham group (n = 3) or the OVX group (n = 3). Rats were anesthetized by intraperitoneal injection of 1% pentobarbital sodium (0.4 mL/100 g). For the OVX group, bilateral ovariectomy was performed. For the sham group, ovaries were exposed but not removed. Successful ovariectomy was confirmed at tissue harvest by gross uterine atrophy in OVX animals compared with sham controls (a standard biological indicator of sustained estrogen deprivation).

### 2.2 Sample preparation for snRNA-Seq

After 3 months of postoperative care, the rats were euthanized with an overdose of pentobarbital sodium (150 mg/kg, intraperitoneally). Death was confirmed by absence of heartbeat and pupillary reflex, followed by cervical dislocation as a secondary physical method in accordance with AVMA guidelines. Immediately thereafter, the lower urinary tract was exposed via a midline abdominal incision. The urethra was dissected from the bladder neck to the external urethral meatus under a stereomicroscope, with careful removal of surrounding connective/vaginal tissues. Samples were rinsed in ice-cold PBS to remove blood/urine and kept on ice throughout processing. For nuclei isolation, urethral tissue was transferred into 1 mL pre-chilled lysate and minced into ∼1 mm pieces. Then, 2 mL of pre-chilled lysate was added, and the suspension was gently homogenized in a Dounce tissue homogenizer (885300–0007; Kimble) for five strokes. The tissue homogenate was incubated on ice for 5 min, followed by addition of 4 mL of pre-cooled 2% BSA dilution. Lysis was terminated by gentle trituration using a wide-bore pipette. Samples were passed through a 30 μm cell strainer, and the filtrate was collected and centrifuged at 300 g for 5 min at 4 °C. The supernatant was discarded. The pellet was washed twice with 2% BSA (300 g for 5 min at 4 °C) and resuspended in 500 µL resuspension buffer (1× PBS, 2% BSA, 0.1% RNase inhibitor). Propidium iodide staining was performed, and debris-free nuclei were sorted using a BD FACSAria II flow cytometer. Viable nuclei were collected and counted.

### 2.3 Chromium 10x genomics library and sequencing

Single-cell nuclear suspension was added to the 10x Chromium chip according to the instructions for the Chromium Next GEM Single Cell 3′Reagent Kits v3.1 (10x Genomics, Pleasanton, CA, United States), with the expectation of capturing 8,000 cells. cDNA amplification and library construction were performed according to standard protocols. Libraries were sequenced by LC-Bio Technology (Hangzhou, China) on an Illumina NovaSeq 6000 sequencing system (double-end sequencing, 150 bp) at a minimum depth of 20,000 reads per cell.

### 2.4 Quality control and cell-type identification

**FIGURE 1:**
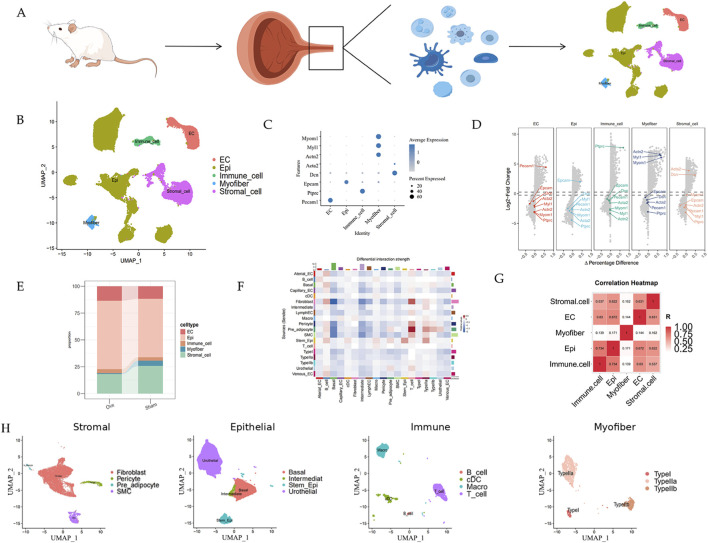
Construction of single-nucleus transcriptome profiles of the rat urethra during menopause. **(A)** Flowchart overview of the experimental design of this study. **(B)** UMAP plot showing major cell types from OVX and Sham rats (subclustering of individual major lineages is shown in Figures 2, 6, 9, 10). **(C)** Dot plot of markers used to annotate major cell types. **(D)** Volcano plot of markers used to annotate major cell types. **(E)** Bar plot of major cell type ratios in different groups. **(F)** Heatmap of cell–cell communication among the major lineages. **(G)** Correlation of DEGs among major cell types. **(H)** UMAP plots summarizing the annotated subpopulations within major lineages (stromal, epithelial, immune, and myofiber compartments).

**FIGURE 2:**
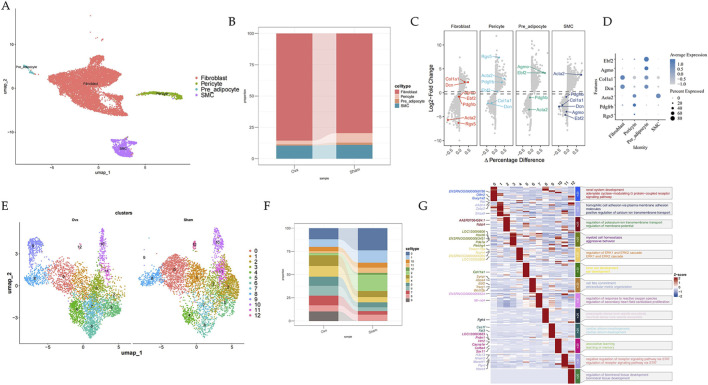
Transcriptional classification of eight subsets of stromal cells and their changes during menopause. **(A)** UMAP plot of stromal cells grouped by cell subpopulations. **(B)** Bar plot showing subpopulation ratios in different groups. **(C)** Volcano plot of markers used to annotate cell subpopulations. **(D)** Dot plot showing markers used to annotate cell subpopulations. **(E)** UMAP plot for fibroblasts grouped by cell subpopulations. **(F)** Bar plot showing subpopulation ratios in different groups. **(G)** Heatmap displaying DEGs for every fibroblast subpopulation and GO enrichment.

Raw sequencing data were processed using the 10x Genomics Cell Ranger pipeline to generate a filtered feature-barcode matrix for each library. Downstream analyses were performed in R using Seurat (version 4.1.0). Nuclei were filtered to remove low-quality profiles and potential doublets. Unless otherwise specified, nuclei with <200 detected genes, >6,000 detected genes, or >5% mitochondrial transcripts were excluded. To mitigate sample-to-sample technical variation, the six libraries were integrated using the Seurat anchor-based workflow (FindIntegrationAnchors/IntegrateData) prior to clustering. Data were normalized (LogNormalize, scale factor = 10,000), and 2,000 highly variable genes were identified. The integrated data were scaled and subjected to principal component analysis (PCA). The top 50 PCs were used to construct the shared nearest neighbor graph (FindNeighbors, dims = 1:50), followed by clustering (FindClusters, resolution = 0.5, Louvain algorithm) and visualization with UMAP. For subclustering of stromal, epithelial, immune, and muscle compartments, the corresponding clusters were subset and re-processed using the same workflow with an empirically chosen resolution (0.6–0.8) based on canonical marker expression. Differentially expressed genes (DEGs) were identified using FindAllMarkers/FindMarkers (Wilcoxon rank-sum test) with min. pct = 0.1 and logfc. threshold = 0.25. P values were adjusted using Bonferroni correction, and genes with adjusted P < 0.05 were considered significant. Cell-type identities were assigned based on canonical marker gene expression and differential expression analysis. Major lineages were annotated using well-established markers (e.g., EPCAM for epithelial cells, COL1A1/DCN for stromal fibroblasts, PECAM1 for endothelial cells, MYOM1/MYL1 for striated myofibers, and PTPRC for immune cells; Figures 1C,D). Each major lineage was then subset and re-clustered to resolve subpopulations using subtype-specific markers (e.g., basal/intermediate/stem/urothelial epithelial cells and B cells/T cells/macrophages/cDC; Figures 2D, 6B, 9C, 10D).

### 2.5 Cell-cell communication prediction and cell cycle analysis

We conducted cell interaction analysis using CellChat (v1.6.1) and CellCall with default parameters (Zhang et al., 2021; Jin et al., 2021). To score the cell cycle phases of every single cell, the Cell_Cycle_Scoring function in Seurat was used based on the expression of canonical marker genes (Nestorowa et al., 2016).

### 2.6 Gene set enrichment analysis

GSEA was performed to examine the significant differences in predefined gene sets within different cell types, which were based on clusterProfiler.

### 2.7 Pseudotime trajectory and RNA velocity analysis

Pseudotime trajectories were inferred using Monocle2 (DDRTree) and Monocle3 as complementary approaches (Cao et al., 2019; Qiu et al., 2017). Specifically, Monocle2 was used to explore an EMT-associated transcriptional continuum between basal epithelial cells and fibroblast subsets, as it provides robust branch-aware trajectory reconstruction. Monocle3 was applied to reconstruct trajectories within the epithelial compartment because of its graph-learning framework that integrates well with UMAP embeddings. We emphasize that pseudotime ordering reflects transcriptomic state relationships and does not by itself prove lineage conversion; therefore, conclusions regarding epithelial-to-mesenchymal plasticity are phrased cautiously. RNA velocity vectors were computed with scVelo (v0.2.4) (Bergen et al., 2020). The dynamical model of transcriptional kinetics was solved by expectation–maximization to estimate reaction rates and latent time for each gene. Velocity vectors were projected onto UMAP and visualized as stream plots.

### 2.8 Identification of co-expressed gene modules

We performed hdWGCNA (version 0.2.19) analysis using default parameters. By calculating the pairwise correlation of the input genes, a topological overlap matrix was obtained after the transformation. kME parameters from the hdWGCNA package were used to define hub genes.

### 2.9 Immunofluorescence staining

Sections were performed with rehydration, antigen retrieval, and blocking, and then incubated with appropriate primary antibodies at 4 °C overnight. Sections were further incubated with secondary antibody for 2 h at room temperature. Nuclei were labeled with DAPI by incubating tissue sections for 10 min. The following antibodies were used: EPCAM (Servicebio, Wuhan), PAX2 (Servicebio, Wuhan), KRT13 (Servicebio, Wuhan), KRT5 (Servicebio, Wuhan), UPK1b (Servicebio, Wuhan), FOS (Servicebio, Wuhan), CD3 (Servicebio, Wuhan), CD68 (Servicebio, Wuhan), MYH1 (Sigma-Aldrich, Germany).

### 2.10 Statistical analyses

All statistical analyses and graph generation were performed in R (version 4.3.2) and GraphPad Prism (version 10.0).

## 3 Results

### 3.1 Overview of the cellular composition of the female rats urethra during menopause

To investigate cellular heterogeneity during menopause, we collected urethral samples from female rats 3 months after bilateral ovariectomy (OVX, n = 3) or sham surgery (Sham, n = 3) and performed snRNA-seq. Gross uterine atrophy was observed in OVX animals at the time of harvest, consistent with successful estrogen deprivation (data not shown). After processing with 10x Genomics and bioinformatics analysis (Figure 1A), the resulting dataset comprises 69,529 nuclei. Five major clusters were identified in the whole cell population based on the expression of known cell type-specific markers, including epithelial cells (Epi), stromal cells, endothelial cells (EC), striated muscle cells (Myofiber), and immune cells (Figures 1B–D). When compared with the sham group, the proportion of stromal cells and striated muscle cells was decreased (Figure 1E). Of note, the stromal cell cluster showed the strongest outgoing signaling capacity among the major lineages (Figure 1F), indicating an active regulatory role of stromal cells in the urethral microenvironment during menopause. The correlation heatmap shows good correlation among the major lineages (Figure 1G). Subsequent analyses were performed by subsetting major lineages and re-clustering to resolve finer subpopulations (e.g., stromal, fibroblast, epithelial, immune, and myofiber subsets; Figures 2, 6, 9, 10). For transparency, we provide an overview UMAP of the annotated subpopulations within the stromal, epithelial, immune, and myofiber compartments (Figure 1H).

### 3.2 Transcriptional classification of fibroblast subsets and their changes during menopause

In this study, we investigate the heterogeneity of stromal cells and the alterations that occur with menopause. Bioinformatics analysis classified all stromal cells into four subsets, which mainly include fibroblasts (FBs), smooth muscle cells, pericytes, and preadipocytes (Figures 2A,C,D). FBs are distributed throughout the stroma of the urethra and are essential for the formation of ECM. Our analysis revealed a significant increase in the proportion of FBs in OVX rats (Figure 2B); yet, the effects of menopause on rat urethra FBs at single-cell resolution remain elusive. To investigate FB cellular changes during menopause at single-cell resolution, we employed an unsupervised visualization approach using the UMAP algorithm, which identified thirteen major cell types. Interestingly, the Fibro09 subset was predominantly present in OVX rats (Figures 2E,F). GO enrichment analysis indicated potential roles for different subsets (Figure 2G).

### 3.3 Cell–cell interaction analysis in the urethral microenvironment under normal and menopausal states

**FIGURE 3:**
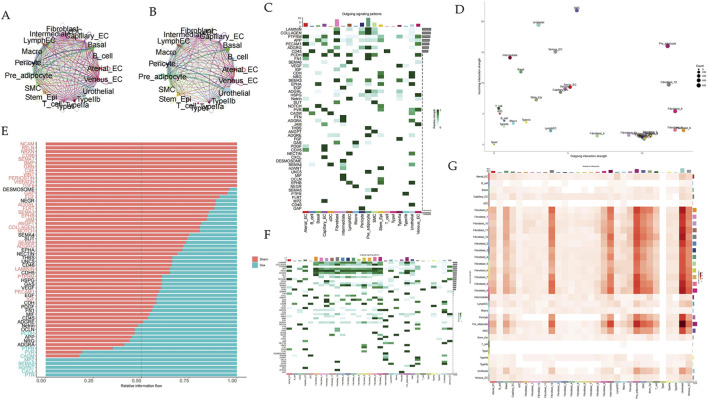
Regulatory signaling of cell–cell communication among the clusters. **(A)** cell–cell communication signaling network among the clusters analyzed in sham rats. **(B)** cell–cell communication signaling network among the clusters analyzed in OVX rats. The width of the lines indicates the number of pairs. **(C)** Heatmap of the CellChat signaling in each cluster. **(D)** Outgoing and incoming interaction strength among each subcluster in OVX group. Cell clusters were located based on the count of their significant incoming (Y-axis) or outgoing (X-axis) signaling patterns. **(E)** The bar plot showing the relative information flow between OVX and Sham group. **(F)** Heatmap of the CellChat signaling in each subcluster. **(G)** Heatmap of cell–cell interactions among the subclusters in OVX group.

To explore the impact of menopause on cellular communication, we compared networks between the sham and OVX groups. Surprisingly, there was a difference in the number and strength of cell-cell interactions in the urethral microenvironment between the two groups (Figures 3A,B). Among the clusters, FBs showed the strongest outgoing signaling pattern in two groups, indicating that FBs were key to regulating the microenvironment’s homeostasis (Figures 3D,G; Supplementary Figures S6A, B). Interestingly, we discovered many classical regulatory pathways (Figures 3C,F). Furthermore, we identified PCDH signals that exhibited different patterns between normal and menopausal states in fibroblasts (Figure 3E; Supplementary Figures S6C, D). These signaling network results helped enhance our understanding of the urethral microenvironment’s physiological and pathological processes.

### 3.4 Basal epithelial cells display EMT-associated transcriptional programs and a potential epithelial-to-mesenchymal state transition in OVX rats

**FIGURE 4:**
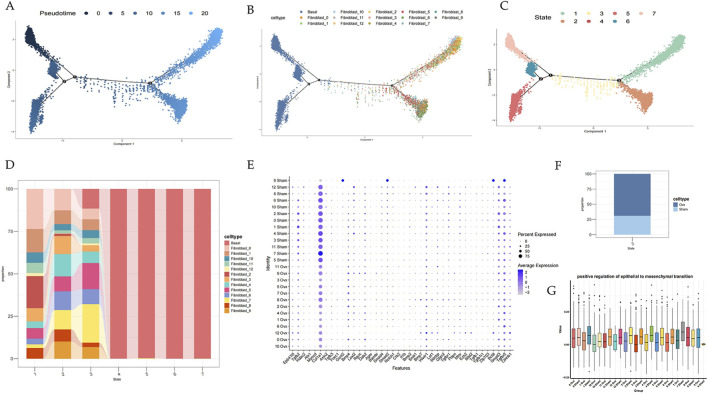
Basal epithelial cells display EMT-associated transcriptional states and a potential epithelial-to-mesenchymal continuum. **(A–C)** Pseudotime analysis of basal epithelial cells and fibroblast subsets. **(D)** Bar plot of cell ratios across seven inferred states. **(E)** Dot plot of EMT-related genes. **(F)** Bar plot showing Fibro09 ratios in different groups. **(G)** Box plot of EMT scores grouped by fibroblast subpopulations.

During menopause we observed a marked shift in stromal composition, with an increased proportion of fibroblasts, particularly the Fibro09 subset, in OVX urethras (Figures 2E,F). Given that epithelial cells can activate EMT-like programs during tissue remodeling (Thiery and Sleeman, 2006; Kalluri and Weinberg, 2009; Kalluri and Neilson, 2003), we asked whether basal epithelial cells may exhibit transcriptional states bridging epithelial and mesenchymal compartments under estrogen deprivation. Therefore, we performed a joint pseudotime analysis of basal cells and fibroblast subsets using Monocle2 to explore an EMT-associated transcriptional continuum (Figures 4A–D). This analysis identified seven states, including a hybrid/intermediate state (State3) that was enriched in OVX samples (Figures 4C,F).

**FIGURE 5:**
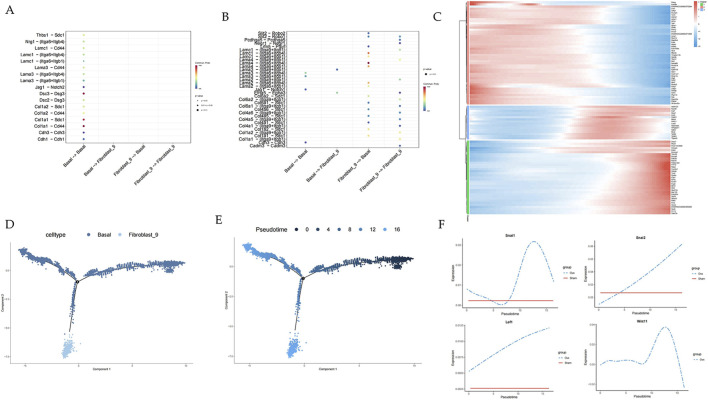
Fibro09 may be associated with EMT. **(A,B)** Dot plot of fibro09-basal ligand-receptor interactions. **(C)** Heatmap of genes changed in 3 clusters. **(D,E)** Pseudotime analysis on fibroblast09 **(F)** Expression of EMT marker genes changed with pseudotime.

Along the inferred pseudotime axis, cells gradually shifted from a basal epithelial transcriptional signature toward mesenchymal-like profiles, accompanied by increased expression of EMT-related regulators and elevated EMT scores, with Fibro09 showing the highest EMT score among fibroblast subsets (Figures 4E,G, 5F). Consistent with this, ligand–receptor analyses revealed a rewiring of basal cell–fibroblast communication in OVX urethras, including enhanced LAMA4/LAMC1–(ITGA6+ITGB4) interactions between Fibro09 and basal cells (Figure 5B), whereas basal cells primarily communicated within the epithelial compartment in Sham urethras (Figure 5A). Collectively, these transcriptomic and interaction patterns suggest that basal epithelial cells activate EMT-associated programs and may acquire mesenchymal-like states under chronic estrogen deprivation. However, pseudotime inference does not establish lineage conversion; future lineage tracing and protein-level validation will be required to conclusively demonstrate epithelial-to-mesenchymal plasticity in the menopausal urethra.

### 3.5 Changes in different epithelial cell subsets during menopause

**FIGURE 6:**
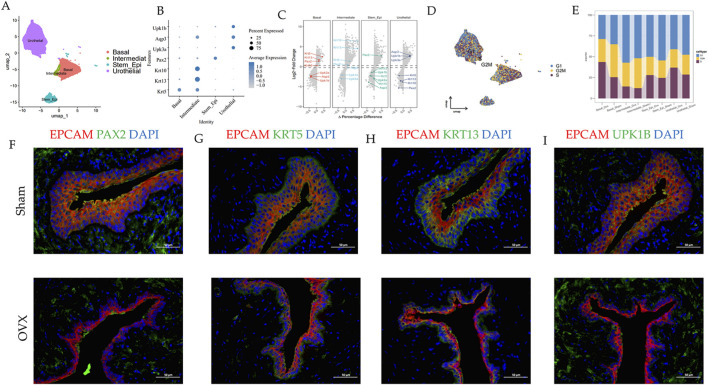
The heterogeneity within the epithelial cluster. **(A)** UMAP plot of epithelial cells grouped by cell subpopulations. **(B)** Dot plot showing markers used to annotate cell subpopulations. **(C)** Volcano plot of markers used to annotate cell subpopulations. **(D)** UMAP plot for three stages (G1, S, G2M). **(E)** Bar plot showing three stages (G1, S, G2M) ratios in different groups. **(F–I)** Multicolor immunofluorescence staining of rats urethra between two groups.

In our data, epithelial cells accounted for the largest proportion of cell types in the urethral tissues (Figure 1E). We further divided the epithelial cells into four subclusters based on gene expression patterns (Figures 6A–C). We then confirmed this result using immunofluorescence staining and found that the deficiency of estrogen leads to atrophy and thinning of the epithelium layer (Figures 6F–I; Supplementary Figures S1A–D; Supplementary Figures S2A–D).

To dissect the cell cycle phases in the urethral tissues, the possible states for each cell cluster were scored using genetic signatures for the G1, S, and G2/M phases. Yet, several clusters had their own patterns. The percentage of each cell cluster in the G1 phase increased in the OVX group. Meanwhile, the percentage of intermediate epithelial cells in the G1 phase also increased in the OVX group (Figures 6D,E).

### 3.6 Fos may be a regulator of epithelial cell communication during menopause

**FIGURE 7:**
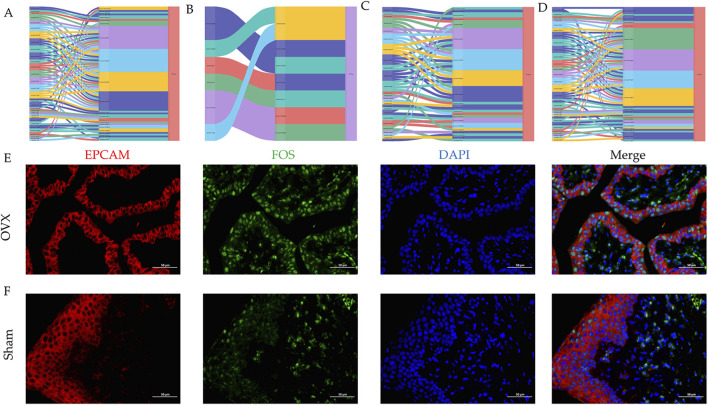
Fos regulates intercellular communication in epithelial cells during menopause. **(A–D)** Sankey diagram of the ligand-receptor-transcription factor in epithelial cell clusters during menopause. **(E,F)** Multicolor immunofluorescence staining of rat urethra between two groups.

In our data, epithelial cells accounted for the largest proportion of cell types in the urethral microenvironment (Figure 1E). To investigate the epithelial cell complex signaling networks, we performed unbiased “ligand–receptor–transcription factor” interaction analyses using CellCall (Figures 7A–D). Interestingly, Fos was predominantly present in the OVX group (Supplementary Figures S3D, E), which was validated by IF (Figures 7E,F). Furthermore, our study demonstrated changes in the communication of potential ligand-receptor pairs between two groups. It was observed that stem cells are primarily receptors in the Sham group and intermediate cells are primarily receptors during menopause (Supplementary Figures S3A, B). Additionally, GSEA and volcano plot revealed that FOS as a transcription factor regulated epithelial cell communication were upregulated during menopause (Supplementary Figures S3C, F–I).

### 3.7 Fos may regulate epithelial cell differentiation during menopause

**FIGURE 8:**
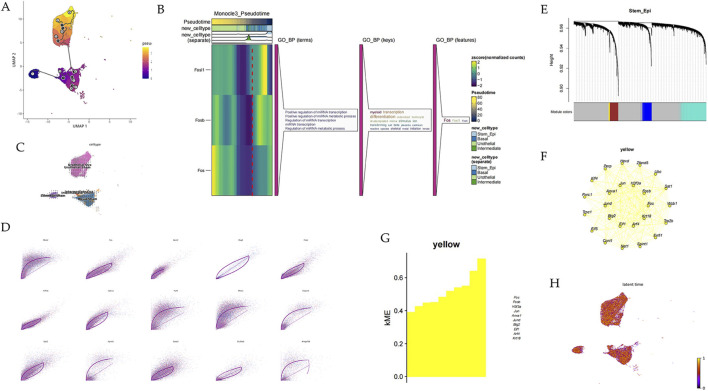
Fos regulates epithelial cells differentiation during menopause. **(A)** Pseudotime analysis on epithelial cells. **(B)** Heatmap of some genes in Module 6. **(C,H)** RNA Velocity Analysis on epithelial cells. **(D)** Fit likelihood top15 genes. **(E)** Gene modules detected through hdWGCNA in stem epithelial cells. **(F)** Fos co-expressed genes network showing **(G)** kME within the yellow module.

Although clustering analysis could reveal heterogeneity among epithelial cells in urethral tissues, it also remains to be determined whether they have common differentiation trajectories. Our pseudo-temporal analysis of all epithelial cell subtypes using Monocle3 revealed a developmental trajectory from stem cells to basal cells, intermediate cells, and urothelial cells (Figure 8A; Supplementary Figures S4B, C, F). We selected genes with significant differential expression and visualized their expression patterns using heatmaps. Interestingly, we found that Module six showed significant differences between the two groups. Module six mainly consists of FOS, FOSB, and some other genes (Figure 8B; Supplementary Figure S4A). To further understand the temporal dynamics of cellular transitions and elucidate the dynamic changes in gene expression and cellular states, we performed RNA velocity analysis in epithelial cells. RNA velocity is a powerful approach that leverages the distinction between spliced and non-spliced mRNA to infer the direction and rate of transcriptional changes in individual cells. RNA velocity analysis revealed distinct dynamic states within the cell populations. By integrating velocity vectors with the UMAP embedding, we visualized the direction and magnitude of transcriptional changes in each cell. The velocity field showed clear directional flows, indicating the progression of cells through different states (Figures 8C,H; Supplementary Figures S4D, E). We identified genes with significant velocity, indicating rapid changes in their expression levels. These genes are likely to be involved in driving cellular transitions; for example, Fos (Figure 8D). In addition, we conducted hdWGCNA to identify gene modules co-expressed with Fos (Figure 8E). We constructed co-expression networks that leading to the identification of 4 gene modules in epithelial stem cells (Figure 8F). The k-Module Membership (kMEs) within the yellow module exhibited the highest overall expression levels within the Fos (Figure 8G). We identified Fos and its co-expressed genes in yellow module. GO analysis revealed that these genes were involved in pathways of cellular response to laminar fluid shear stress (Supplementary Figures S4G–I).

### 3.8 T cells from OVX rats have a stronger inflammatory response

**FIGURE 9:**
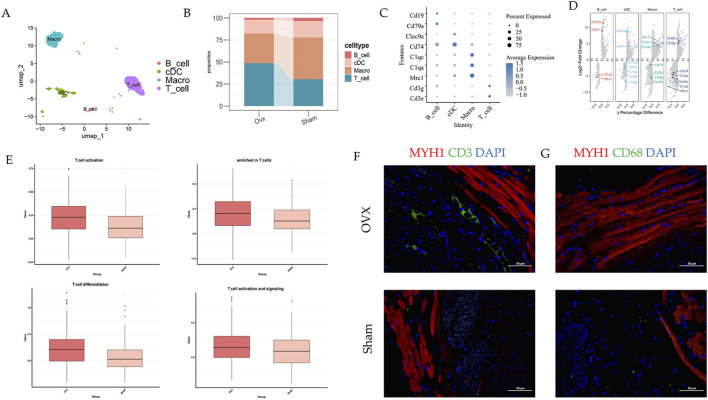
T cells from OVX rats have a stronger inflammatory response. **(A)** UMAP plot of immune cells grouped by cell subpopulations. **(B)** Bar plot of immune cell subpopulation ratios in different groups. **(C)** Dot plot showing markers used to annotate cell subpopulations. **(D)** Volcano plot of markers used to annotate cell subpopulations. **(E)** Chronic inflammatory response gene set scores of T cell subpopulations **(F,G)** Immunofluorescence staining of T cells and macrophages.

We also conducted further clustering and analysis of immune cells. Based on their respective marker genes, we categorized immune cells into four subsets (Figures 9A,C,D). It is noteworthy that the increased ratio of T cells and decreased ratio of macrophage in OVX group (Figure 9B). We then confirmed this result using immunofluorescence staining (Figures 9F,G; Supplementary Figures S5A–D). Furthermore, we examined the scores of inflammation and inflammatory response in various subsets (Figure 9E). In summary, T cells from OVX rats exhibited a more pronounced inflammatory response.

### 3.9 Tmem233 may be crucial for the differentiation and functional maintenance of type II striated muscle fibers

**FIGURE 10:**
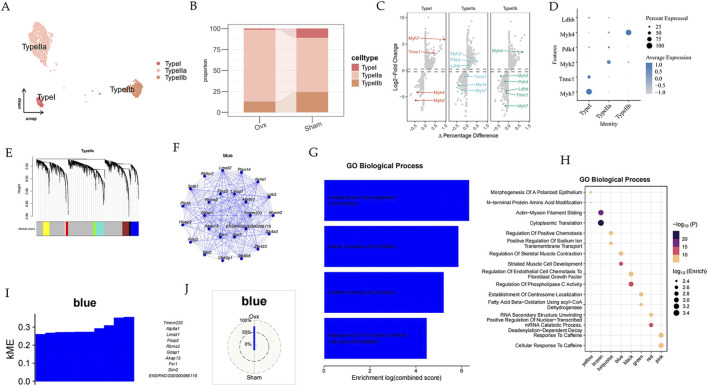
The characteristics of myofiber clusters during menopause. **(A)** UMAP plot of immune cells grouped by cell subpopulations. **(B)** Bar plot of immune cell subpopulation ratios in different groups. **(C)** Dot plot showing markers used to annotate cell subpopulations. **(D)** Volcano plot of markers used to annotate cell subpopulations. **(E)** Gene modules detected through hdWGCNA in type II striated muscle cells. **(F)** Co-expressed gene network within the blue module. **(G,H)** GO analysis (biological process) shown as a bubble diagram. **(I)** kME within the blue module. **(J)** The Radar Plot shows the percentage distribution of the blue modules in different groups.

We further divided the myofiber into three subclusters based on gene expression patterns (Figures 10A–D). Urethral pressure is mainly generated by muscle contraction, and its structural and functional disorders are closely related to urinary incontinence, especially type II a. We conducted hdWGCNA to compare the differential genes and functions between the two groups. Furthermore, we constructed co-expression networks that led to the identification of 8 gene modules (Figure 10E). GO analysis revealed that the blue module was involved in pathways of Regulation of skeletal muscle contraction and striated muscle cell development (Figures 10G,H,J). kMEs within the blue module exhibited high expression levels of Tmem233 (Figure 10I). We identified Tmem233 and its co-expressed genes in the blue module (Figure 10F).

## 4 Discussion

Menopause-associated estrogen deficiency is linked to a high prevalence of lower urinary tract symptoms, including urinary urgency/frequency, recurrent urinary tract infections, and stress urinary incontinence (Robinson et al., 2013). These symptoms are thought to arise from multi-compartment remodeling of the urethral epithelium, stroma, muscle, and immune microenvironment, yet the cell-type-resolved molecular basis remains poorly defined (Alperin et al., 2019). Here, we generated a snRNA-seq atlas of the female rat urethra in sham and OVX-induced hypoestrogenic conditions, providing a framework to map estrogen-responsive programs at single-cell resolution and to nominate candidate pathways and cell populations for regenerative or cell-based strategies (Wang et al., 2023).

We identified five major urethral cell lineages and resolved multiple stromal and epithelial subtypes. Cell–cell communication analyses highlighted fibroblasts as a dominant source of outgoing signals in both sham and OVX urethras, with menopause altering the strength and composition of communication networks. These findings support an active regulatory role of stromal fibroblasts beyond extracellular matrix production and are consistent with reports that estrogen deficiency remodels collagen and ECM in the lower urinary tract (Zhang et al., 2024).

Within the epithelial compartment, we found increased Fos activity in OVX urethras and identified Fos-associated transcriptional modules linked to epithelial communication and differentiation. Fos/AP-1 is an immediate early transcription factor responsive to mechanical stress and inflammatory cues and can regulate epithelial proliferation and barrier homeostasis (de Groat, 2013; Huhe et al., 2017; Jafari and Rohn, 2022; Preston et al., 1996). Our data suggest that chronic estrogen deprivation engages a Fos-centered regulatory program, potentially contributing to altered epithelial cell states during menopause.

Joint analyses of basal epithelial cells and fibroblast subsets revealed an EMT-associated transcriptional continuum and a hybrid state enriched in OVX urethras. Together with elevated EMT scores and rewired basal–fibroblast signaling, these results are consistent with EMT-like plasticity contributing to stromal remodeling. Because trajectory inference is hypothesis-generating, the possibility of true epithelial-to-mesenchymal conversion requires further validation using lineage tracing, spatial approaches, and protein-level assessment of epithelial and mesenchymal markers (Thiery and Sleeman, 2006; Kalluri and Weinberg, 2009; Kalluri and Neilson, 2003; Lamouille et al., 2014).

In the immune compartment, OVX urethras exhibited a higher proportion of T cells and stronger inflammatory response signatures, which may contribute to chronic local inflammation in the postmenopausal urethra (Shen et al., 2024; Xu et al., 2025). In the muscle compartment, module-based network analysis nominated Tmem233 as a hub gene in a contraction-related program in type IIa myofibers, suggesting a potential link between estrogen deprivation and altered urethral muscle maintenance (Andersson and Uvelius, 2024).

Several limitations should be acknowledged. First, OVX rats recapitulate chronic estrogen deprivation but do not fully model human menopause. Second, direct physiological readouts relevant to continence (e.g., leak point pressure) were not assessed in this cohort, and serum estradiol/quantitative uterine indices were not measured; these assessments will be important in future work to link molecular programs with functional outcomes. Third, sample size was modest (n = 3 per group), and key computational inferences (cell–cell communication, trajectory analysis, and RNA velocity) require independent experimental validation.

## 5 Conclusion

In conclusion, we performed an snRNA-seq analysis of the urethra from female rats. Our data reveal the transcriptome landscape and cellular heterogeneity of the female rat’s urethra. By elucidating the differentiation development trajectory of cell populations and their interactions, our study contributes to a better understanding of the dynamic changes in the microenvironment of the urethra during menopause. Future studies focusing on the consequences of these estrogen changes of urethra may pave the way for the development of novel therapeutic strategies.

## Data Availability

The raw data supporting the conclusions of this article will be made available by the authors, without undue reservation.
