# Supplementary material for: Unveiling urethral cellular heterogeneity in menopause through single-nucleus RNA sequencing
Source: Front Physiol. 2026 Mar 2;17:1753680. doi: 10.3389/fphys.2026.1753680 (PMC12989360; doi:10.3389/fphys.2026.1753680)
Supplement: Supplementary file 1 [file DataSheet1.docx]

**Supplementary Figures**


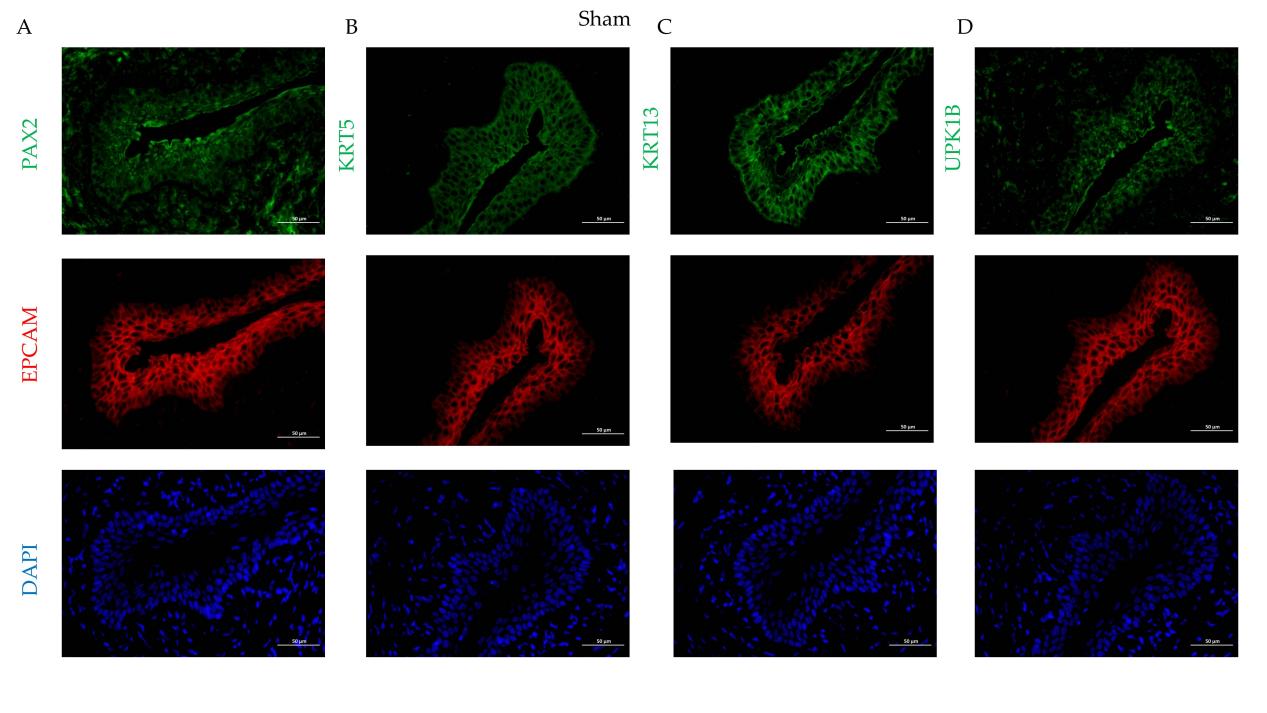


**Supplementary Fig. 1** (A) Immunofluorescence staining of stem epithelial cells in the Sham group. (B) Immunofluorescence staining of basal cells in the Sham group. (C) Immunofluorescence staining of intermediate cells in the Sham group. (D) Immunofluorescence staining of urothelial cells in the Sham group.


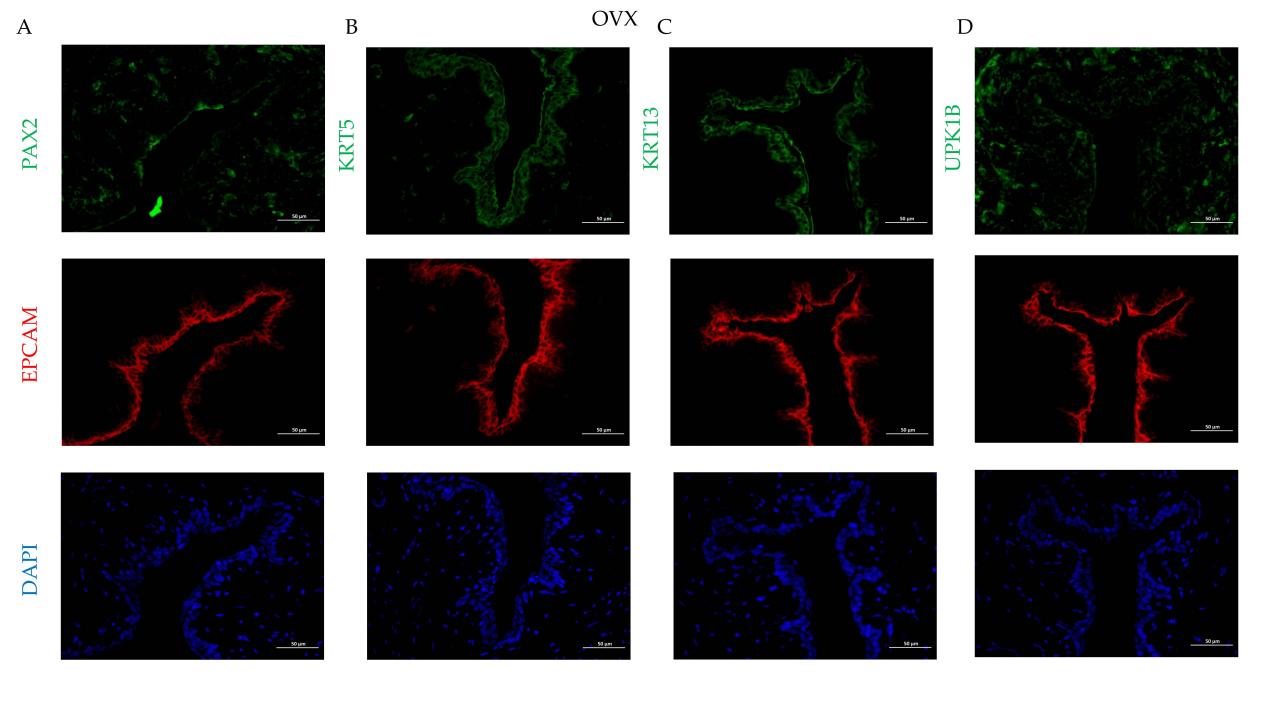


**Supplementary Fig. 2** (A) Immunofluorescence staining of stem epithelial cells in the Sham group. (B) Immunofluorescence staining of basal cells in the Sham group. (C) Immunofluorescence staining of intermediate cells in the Sham group. (D) Immunofluorescence staining of urothelial cells in the Sham group.

**
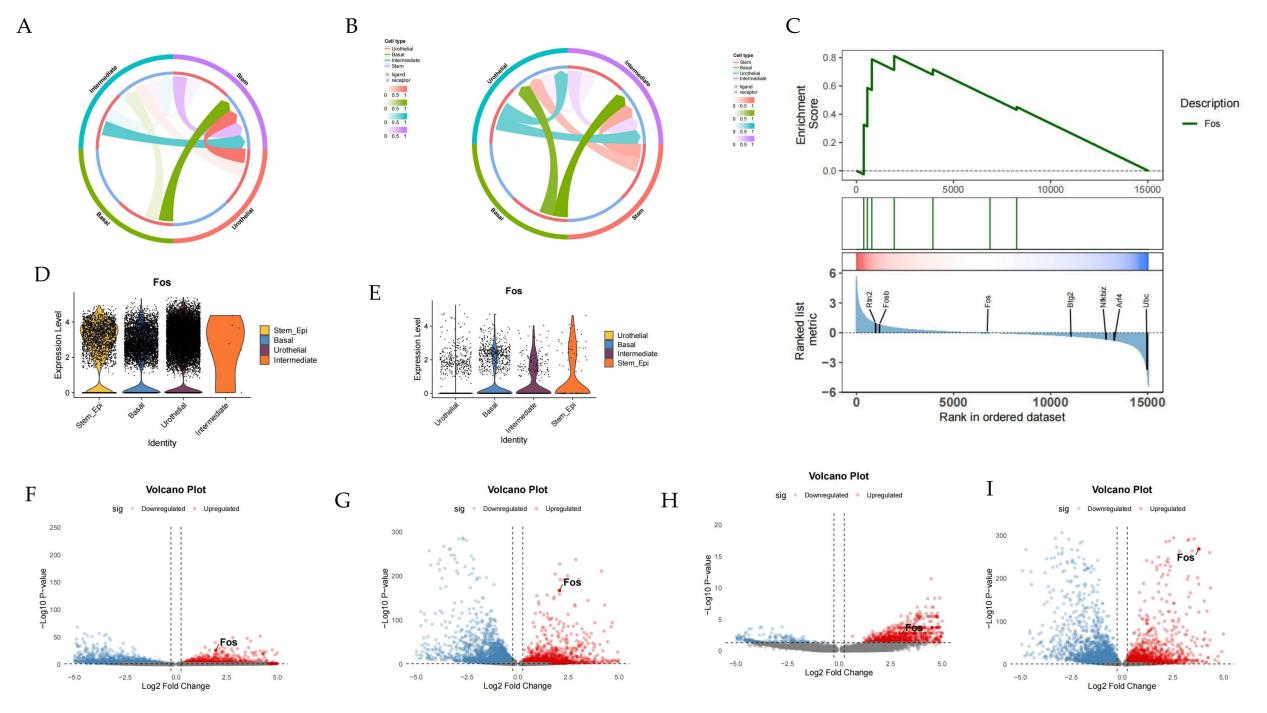
**

**Supplementary Fig. 3** (A) Circos diagram of ligand-receptor interactions in the Sham group. (B) Circos diagram of ligand-receptor interactions in OVX group. (C) GSEA analysis of Fos. (D) Expression level of Fos in OVX group. (E) Expression level of Fos in the Sham group. (F) Volcano plot of Fos stem epithelial cells.

**
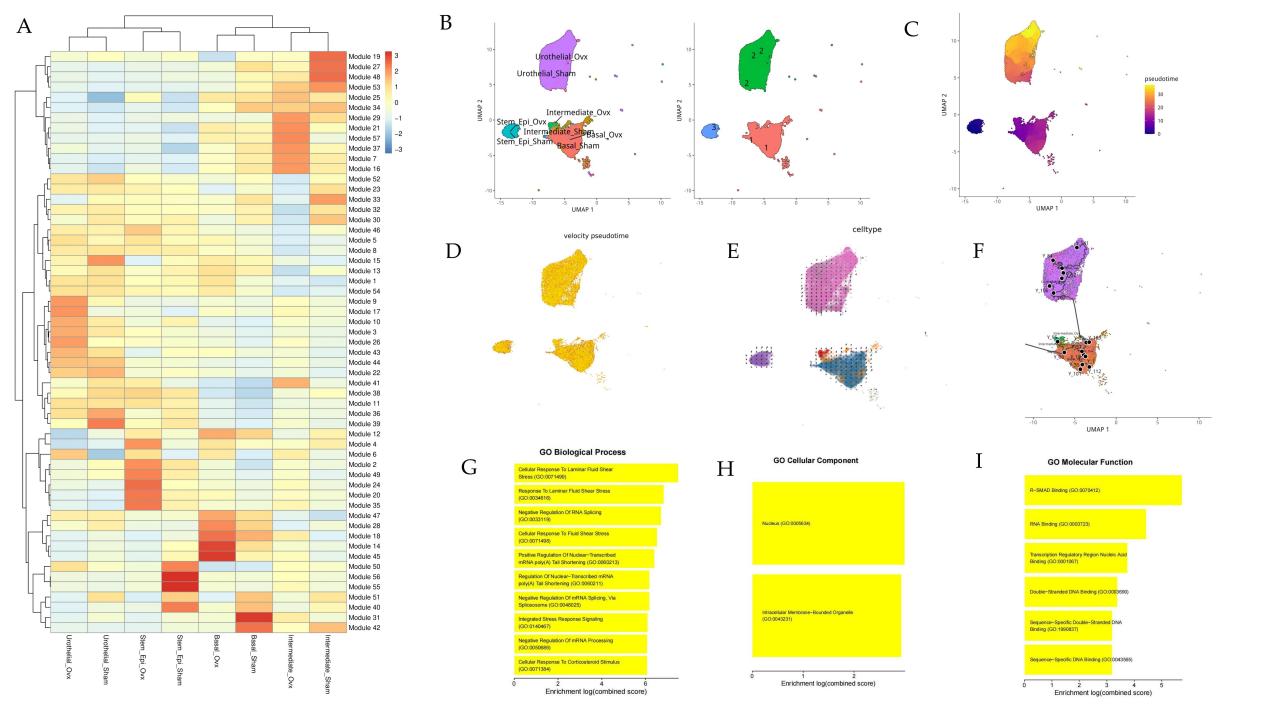
**

**Supplementary Fig. 4** (A) Heatmap of Pseudotime analysis; (B,C,F): Monocle3-based pseudotime trajectory of epithelial cells; (D,E): RNA Velocity Analysis on epithelial cells. (G-I) GO Enrichment analysis

**
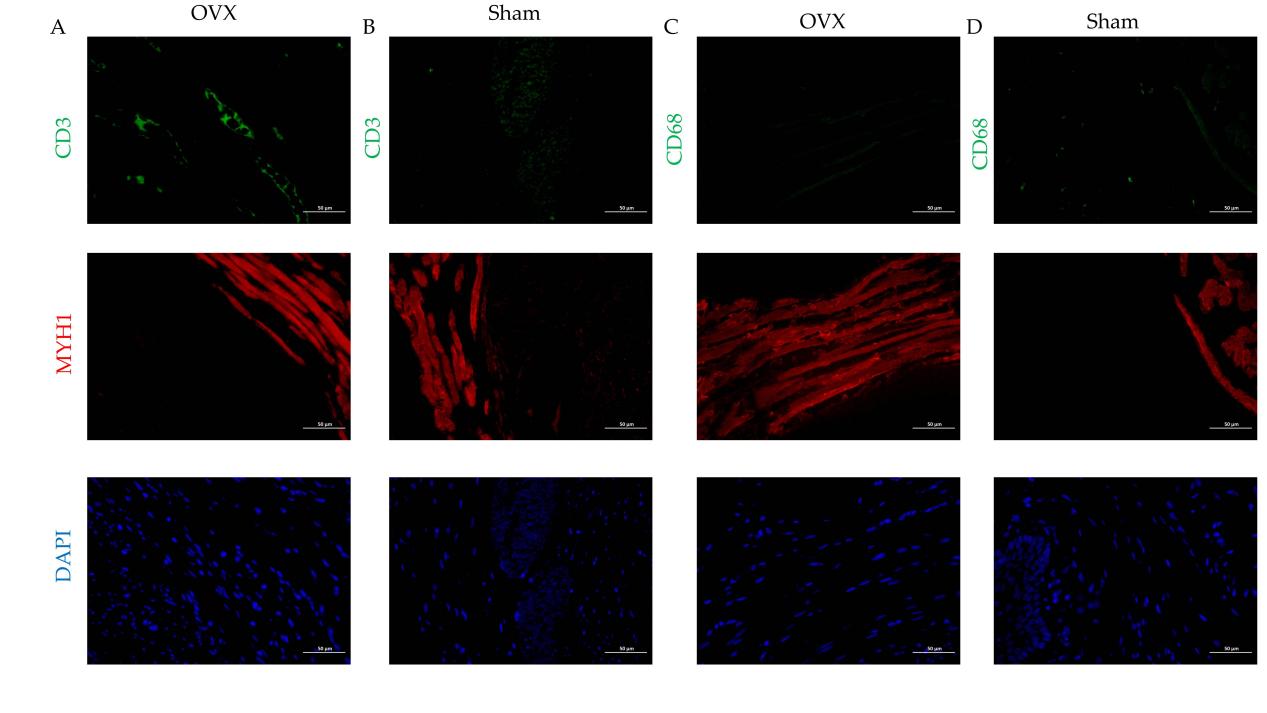
**

**Supplementary Fig. 5** (A) Immunofluorescence staining of T cells in OVX group;(B): Immunofluorescence staining of T cells in the Sham group;(C): immunofluorescence staining of macrophages in OVX groups;(D): immunofluorescence staining macrophagesin sham groups.

**
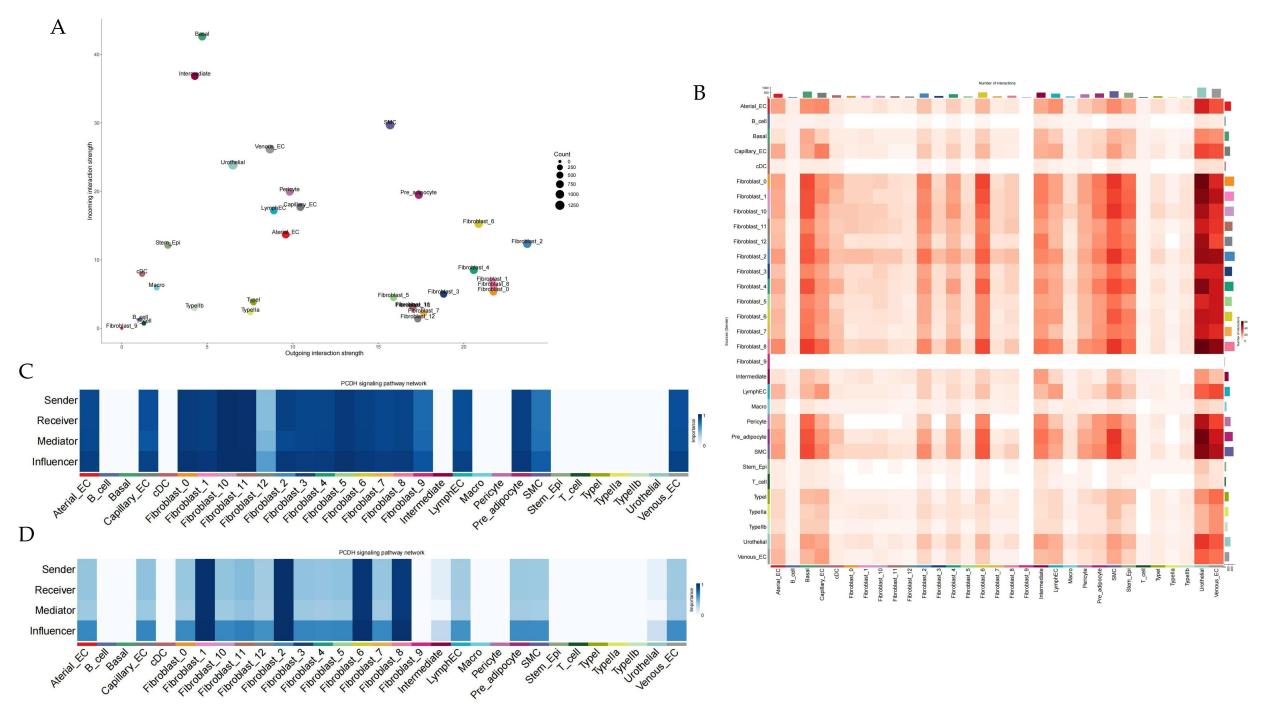
**

**Supplementary Fig. 6** (A). Outgoing and incoming interaction strength among each subclusterin sham group. Cell clusters were located based on the count of their significant incoming (Y-axis), or outgoing (X-axis) signaling patterns. (B) Heatmap of cell–cell interactions among the subclusters in Sham group. (C) PCDH signaling pathway network in OVX group. (D) PCDH signaling pathway network in Sham group.
